# A Demand-Centric Repositioning Strategy for Bike-Sharing Systems

**DOI:** 10.3390/s22155580

**Published:** 2022-07-26

**Authors:** Ying-Chih Lin

**Affiliations:** Department of Applied Mathematics, Feng Chia University, Taichung 407102, Taiwan; yichlin@fcu.edu.tw

**Keywords:** bike sharing, repositioning, clustering, IoT, smart city

## Abstract

Transport-sharing systems are eco-friendly and the most promising services in smart urban environments, where the booming Internet of things (IoT) technologies play an important role in the smart infrastructure. Due to the imbalanced bike distribution, bikes and stalls in the docking stations could be unavailable when needed, leading to bad customer experiences. We develop a dynamic repositioning strategy for the management of bikes in this paper, which supports dispatchers to keep stations in service. Two open datasets are examined, and the exploratory data analysis presents that there is a significant difference of travel patterns between working and non-working days, where the former has an excess demand at rush hours and the latter is usually at a low demand. To evaluate the effect when the demand outstrips a station’s capacity, we propose a non-linear scaling technique to transform demand patterns and perform the clustering analysis for each of five categories obtained from the sophisticated analysis of the dataset. Our repositioning strategy is developed according to the transformed demands. Compared with the previous work, numerical simulations reveal that our strategy has a better performance for high-demand stations, and thus can substantially reduce the repositioning cost, which brings benefit to bike-sharing operators for managing the city bike system.

## 1. Introduction

One of the booming Internet of things (IoT) and Internet of everything (IoE) application domains is smart cities [1], where an agile, collaborative and sustainable smart city ecosystem delivers livable, attractive and resource-efficient cities. A cost-sharing mechanism is a major element of smart cities by tackling some of the imperative urban challenges such as energy use, carbon reduction and reuse of materials. The practice of sharing transport (cars, bikes, etc.) is not only a sustainable practice but also could establish better human liaisons in the so-called sharing city [2], which is a concept that emerged recently as a new notion for urban development. A bike-sharing system (BSS) allows people to borrow bikes from stalls in a station and return them at another station with the same system. It is conceivable that keeping the system in service is challenging since the dynamic human mobility often causes the inevitable imbalance between available bikes and stalls. A key to success for the system is the efficient repositioning operations, that is, refilling a station before the supply runs dry and removing bikes from the fully occupied station.

There has been a great deal of works to study related issues of BSS. In the station analysis, the imbalance between rental and return demands is ubiquitous for each bike station, where probable reasons include population density, demand period, weather and so on [3]. An area of high population density (e.g., colleges and MRS stations) can have high rental and return demands [4]; the weather condition is also factor that affects the bike-sharing demand, where rainy days have a lower demand than sunny days [4,5] and the demand in summer is higher than in the winter [5]; the working day can have the peak demand during the rush hour, whereas the demand is much lower during the weekend [6,7]; the proportion of commuting trips is much lower during the COVID-19 pandemic [8]. In addition, stations have diverse demands in various influence factors, while the clustering analysis of stations can bring benefits to bike-sharing operators that contribute to an efficient repositioning and maintenance system. Common methods of the station clustering in previous works are hierarchical clustering [9,10], *k*-means [11,12], DBSCAN [13,14], etc. Bordagaray et al. [15] classify bike-share demand into five usage behaviors through data mining techniques: round trips, rental time reset, bike substitution, perfectly symmetrical mobility trips and non-perfectly symmetrical mobility trips. A study to analyze travel patterns of BSS usually ignores the round trips made by short activities in the first usage type and very brief dwell times of the third usage type.

In addition, some studies focus on the prediction of rental and return demands for stations, while common models in recent years include multiple regression analysis [16], random forest [17,18], boosting framework [17,18,19], deep learning [18,20], etc. Several works employ probability distributions to model the number of trips at each station, containing negative binomial [17,21], Weibull [22,23] and Poisson [16,17], where the latter is often the best choice for this task. The demand forecasting can encourage operators to grasp the urgent bike/stall demand of stations and reallocate bikes accordingly. Actual circumstances to reposition bikes are so complicated, including trunk capacity, repositioning route, manpower deployment, etc., that most works focus on studying one or some issues. To facilitate the intelligent management of BSS, Alaoui and Tekouabou [24] integrate both the IoT for smart city technologies and machine learning to develop an automatic management system capable of forecasting the user demand in real-time.

The repositioning operation in BSS moves bikes across different stations by trucks usually to satisfy customers’ demand. During the day, the bike-sharing operator may use trikes or corrals instead for vehicular convenience and efficiency. A trike is a type of trailer that can hold a very limited number of bikes and is towed by a cyclist to dispatch bikes, whereas a corral offers an on-street bike parking space of small size and is contained within a regular car parking stall. Both the options allow more flexible repositioning techniques. Previous works tackle the BSS repositioning problem by either static or dynamic manners. A static strategy relocates bikes at routine times or when both traffic and demand are low, e.g., during night time, whereas a dynamic one works once a station is going to be unavailable, i.e., full or empty station. Obviously, the static repositioning is hardly content with frequent rental and return demands; however, it can be modeled as optimization problems whose objective is to route trunks of finite capacity to meet station targets while minimizing the route length [25,26].

Compared to the static bike repositioning, the dynamic strategy timely reallocates bikes so that stations stay available, although it has many challenges such as station status prediction, station clustering with common demand type and repositioning path planning. Contardo et al. [27] firstly modeled the dynamic repositioning problem as an optimization problem on the complete directed graph, and then proposed two decomposition schemes to obtain feasible solutions in tractable time. Based on the demand estimation by a stochastic process, e.g., Poisson [16,17] and Markov chain [28,29], some works can significantly reduce the complexity of the prediction problem, where Liu and Pelechrinis [16] adopt the Poisson regression and Skellam regression to estimate the excess demand of bikes or stalls. Chiariotti et al. [6] give an alternative route of the Birth–Death process to model stations’ occupancy and estimate the amount of time a station is unavailable, i.e., either empty or full. Vallez et al. [30] propose a thorough review to point out challenges and opportunities behind the bike repositioning, while various repositioning frameworks continue to be presented with experiments on real data of different locations for the performance evaluation [31,32]. More recently, the repositioning problem has been reformulated as a mixed-integer nonlinear model so that its linearization model can be solved efficiently [14]. On the other hand, several works exhibit the time series periodicity in historical bike-sharing demand by examining real datasets [33,34].

This study develops a repositioning scheme based on the historical travel trips obtained by two open datasets, Citi Bike trip and transportation data. The exploratory data analysis gives some insights into demand patterns and we sophisticatedly group the trip times into four categories derived from two types of days in two seasons. To highlight the excess demand for stations, we utilize a non-linear scaling technique to standardize the rental and return demands and perform the clustering analysis to group stations with similar demand patterns. Compared with the previous work, our repositioning scheme has outstanding performance through simulated experiments and several measurements.

The rest of this paper is organized as follows: Section 2.1 presents the sources of two datasets examined further in Section 2.2. Section 2.4 introduces the station clustering based on the non-linear standardization of demand patterns in Section 2.3. Subsequently, our dynamic repositioning strategy is introduced in Section 2.5. We conduct several experiments to evaluate the performance, where Section 3.1 demonstrates and summarizes the clustering result. The pseudocode of our repositioning strategy is shown in Section 3.3 and is compared with the previous work using measurements of Section 3.2. Finally, Section 4 draws our conclusions.

## 2. Materials and Methods

### 2.1. Data Source

There are two datasets in this work: One is the bike ride data released by the Citi Bike official site [35]; the other is the temporal dataset of geolocated bikes from New York City (NYC) [36]. Citi Bike is privately owned and is the largest BSS with 24,500 bikes and over 1500 stations serving several boroughs of NYC, and its trip data are a popular material for extensively exploring the BSS. It began operations in May 2013 and, in June of the same year, published the historical trip data on its website. This dataset has been processed to remove unconventional trips (e.g., trips taken by staff for service and engineers for testing) and any trips whose length is below 60 s (e.g., bike substitution). The trip with a short period potentially results from riding by false starts or trying to re-dock a bike, which are similar to the rental time reset and bike substitution, respectively [15]. We use the four features, i.e., Start/Stop Time and Date, Start/End Station, to represent a trip.

The other dataset is the transportation data initially collected to explore the relationship between policy intervention, bus service quality and changes in commuter mode share [36]. The dataset includes trip records from March 2015 to April 2019 to provide retrospective data and summary statistics and is available online [37]. Records are captured approximately every 10 min, and perhaps are incomplete due to interruptions in the communication infrastructure, which are rare and can be easily processed. We use the eight features, i.e., dock_id, date, hour of day, minute, pm (0:am,1:pm), avail_bikes, avail_docks and tot_docks. Since two datasets have different time periods, here we select stations with complete trip records over the period of 2018 in both datasets, which include 627 stations in total. In addition, we resample each record by 30 min and simply impute missing values by the mean of two neighbor values.

### 2.2. Exploratory Data Analysis

**Figure 1:**
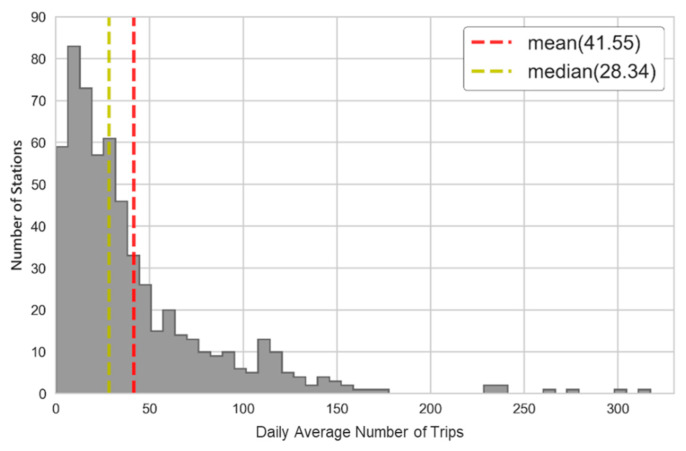
Daily average number of trips for individual station.

First, we illustrate the average of the daily number of trips for each individual station, as seen in Figure 1, and there is a long tail on the right of the distribution, i.e., positive skew (or right skew). The mass of the distribution is mostly concentrated on the left of the figure, leading to a much larger mean than median. Most stations are underutilized, but there are few stations in high-capacity demand. In a word, the top 5% capacity demand is around two times greater than the mean, implying the high demand of minority stations.

**Figure 2:**
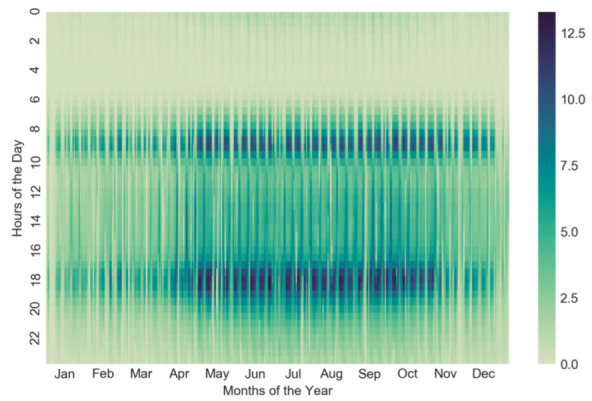
Average of aggregated number of trips for all stations.

**Figure 3:**
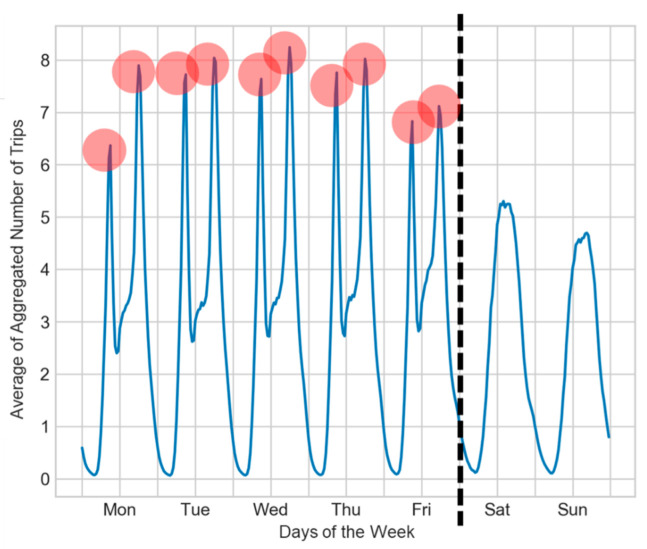
Daily average of aggregated number of trips in a week, where the blue line is the average demand, weekdays and weekends are separated by the black dotted line, and red circles represent brief peak demands.

Figure 2 is a heat map showing the average of the aggregated number of trips every 30 min for all stations, where darker colors indicate higher demand. In addition to the higher demand between May and October, Figure 2 demonstrates two darker bands near 9 a.m. and 6 p.m. with a high demand. Dark and light colors within the two bands are interlaced, where light colors appear every short period of time, which is likely due to the effects of working and non-working days, as shown by Figure 3.

**Figure 4:**
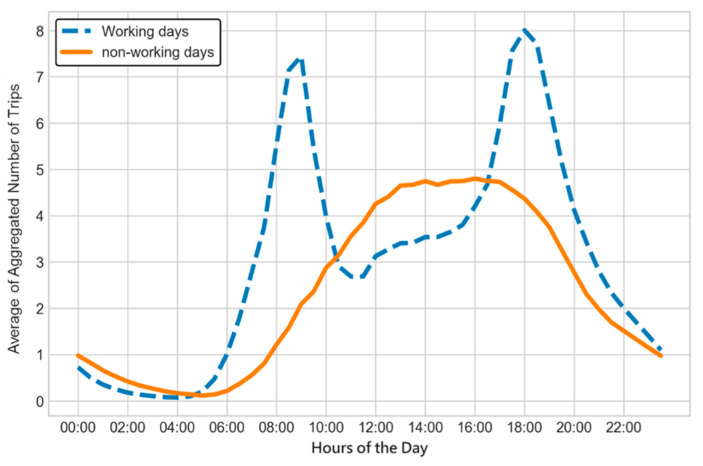
Average demands of working and non-working days.

For the insight from Figure 2, we illustrate the average demand in a day for all stations with respect to the working and non-working days, seen in Figure 4. Two different types of days are defined by the Office Holidays website [38], where the working days shown by the blue-dotted line in Figure 4 are the days except weekends and holidays. Two patterns of demand in the figure are quite distinct. From Figure 4, there are two peaks, i.e., 9 a.m. and 6 p.m., on working days, and a station has 3.053 rental and return demands (RRDs) every 30 min on average; however, the curve of non-working days is relatively smooth and has a high demand in the afternoon, where its average demand is 23% lower than the working days. Though there is a major distinction between the two patterns, they are both in low demand from 0 to 5 a.m. Overall, the curve of RRDs on non-working days is smoother and has a lower demand than working days.

### 2.3. Demand Scaling

**Figure 5:**
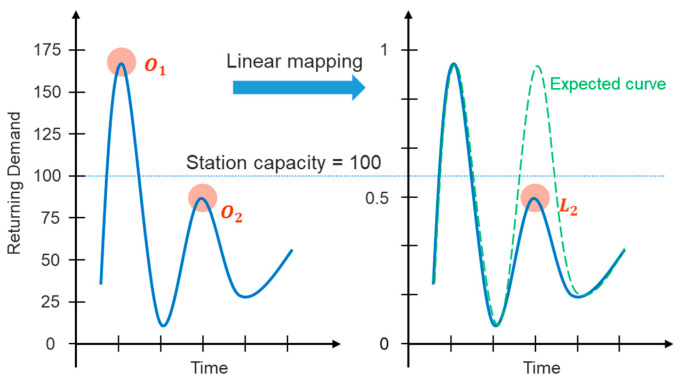
Diagram for linear mapping on returning demand.

Owing to the peak demand on working days and smooth demand on non-working days, it is arduous to accurately present the renting and returning state at every time for each station. Therefore, this work proposes a *demand scaling* (DS) technique to standardize the demand by mapping it to [0, 1]. Such an idea tries to precisely reveal the demand tolerance for each station with a different capacity. By intuition, a station of larger capacity can afford greater RRDs, whereas a small-sized station has a narrow range. However, thanks to reallocating bikes of an in-service station, it could have a greater upper bound of demand over a period of in-service time. The DS transforms the station demand into the interval of [0, 1] based on the maximum capacity of the station, which can respond to the real strength of demand. On the other hand, conventional linear transformations may not reveal the peak demand in practice. Take Figure 5 as an example, the left figure exhibits the raw demand, whereas the solid line in the right one is the corresponding demand after a linear transformation and the dotted line represents the expected result, where a great demand relative to the station capacity is approaching 1. Figure 5 assumes the station capacity is 100, and the line is the cumulatively returning demand every 30 min, whose value can be larger than the station capacity due to continuous renting or dynamic repositioning. In the *O*_1_ of Figure 5, its demand far exceeds the station capacity and thus it represents the time of frequent returns; however, the demand in the *O*_2_ is extremely high despite the lower value than the station capacity. Applying linear mapping (e.g., min-max scaling) on the raw demand maps the demand in *O*_1_ and *O*_2_ to 1 and *L*_2_ respectively, where we are likely to underestimate the high demand in *O*_2_.

To tackle the above issue, we develop a non-linear mapping called *demand scaling* (DS), attempting to transform the relatively high demands in *O*_1_ and *O*_2_ into near 1. The following are formulas *DS_rent_* and *DS_return_* to transform two types of demand where $\mu_{s}\left(t_{i}\right)$ and $\lambda_{s}\left(t_{i}\right)$ represent the RRDs in time *t_i_*, respectively. *C_s_* in the formula is the capacity of station *S*, while the given constant *c* > 0 controls the rate of approaching 1 with a small *c* making the convergence fast.

(1) $$DS_{rent}\left(C_{s},\;t_{i},c\right)=1-\frac{2}{1+{\left(\mu_{s}\left(t_{i}\right)+1\right)}^{\frac{\mu_{s}\left(t_{i}\right)}{C_{s}}\times \frac{1}{c}}}$$

(2) $$DS_{return}\left(C_{s},\;t_{i},c\right)=1-\frac{2}{1+{\left(\lambda_{s}\left(t_{i}\right)+1\right)}^{\frac{\lambda_{s}\left(t_{i}\right)}{C_{s}}\times \frac{1}{c}}}$$

**Figure 6:**
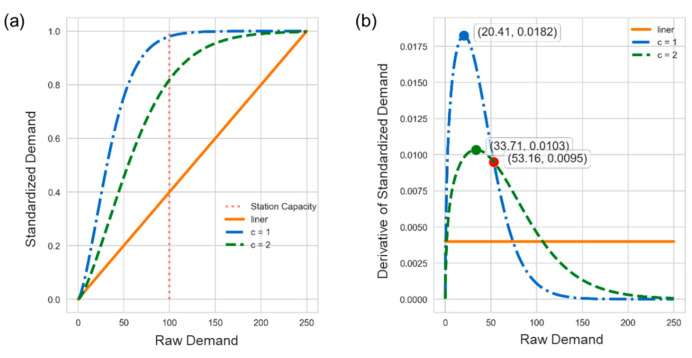
(**a**) Transformed results under different *c* values; (**b**) Slopes of curves in (**a**).

**Figure 7:**
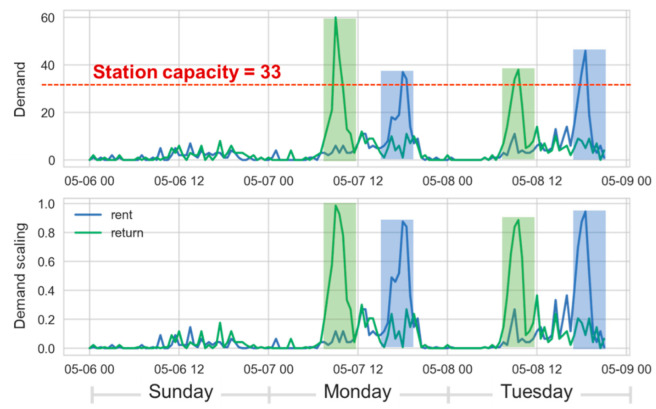
Comparison of the demand in a real situation and its standardization by demand scaling.

Figure 6a compares transformed results on the demand under different *c* values where the station capacity is 100. In contrast to the linearly transformed curve shown by the orange and solid line, the standardized demands of two non-linearly transformed curves can give the actual state of demand by virtue of approaching 1 at the station capacity. Moreover, Figure 6b demonstrates the corresponding slopes of curves in Figure 6a with the same color and style of lines. The maximal slope of blue curve with *c* = 1 (20.41) is slightly smaller than that of green curve with *c* = 1 (33.71), but the latter is smoother than the former. The value *c* can be somehow regarded as the level of tolerance for the maximal renting/returning demand of a station, where a small *c* is more sensitive to the demand and skyrockets at low demand times. Take a real station with a capacity of 33 as an example in Figure 7, the green curve is the returning demand and two green blocks indicate the relatively high demand. After the DS standardization, two returning curves in green blocks are both close to 1, making the level of demand stand out against the three days. Likewise, there are two peaks in the blue line and blocks.

### 2.4. Station Clustering

Stations may have distinct types of demand due to weather, holiday, service hours, etc., and correctly identifying the demand types of stations would facilitate the dynamic repositioning methodology for bike-sharing systems. A popular method to determine the demand type is by clustering algorithms, where stations in the same cluster are regarded as having a similar demand type. A station is represented by a feature vector of extracting valuable information from the dataset, such as location, capacity as well as renting/returning type, and it helps the clustering algorithm to understand and work accordingly. Here, we adopt the date and station demand to form the feature vector. Moreover, Figure 2 and Figure 4 exhibit the significant differences in the station demand between dull (January–April, November and December) and peak (May–October) seasons as well as between working and non-working days, respectively.

Where the station demand varies considerably over time from Figure 2 and Figure 4, we sophisticatedly divide the trip times into four categories corresponding to the four combinations of two seasons and two days. For each category, the hourly average demand is computed for every station and thus the station demand is represented by 48 features with each demand within 30 min. Then, the DS is applied to each feature and standardizes it to [0, 1] for accentuating the high demand and subsequent clustering. To exactly determine the demand type of a station, we adopt the classical *k*-means algorithm to partition stations in a category into *k* clusters, i.e., *k* demand types. Each station belonging to the cluster with the nearest mean (also named cluster center or centroid) serves as a prototype of the cluster. The silhouette coefficient to measure the internal cluster and the elbow illustration to the explained variation are utilized at determining an appropriate *k*.

### 2.5. Repositioning Strategy

The dynamic repositioning strategy in this work is based on the so-called *decision interval* consisting of low, targeted and upper values for a station. The primary idea of our repositioning strategy is to adjust the number of available stalls to the targeted value in case it is beyond the lower and upper values. Consequently, the *demand-centric repositioning strategy* (DCRS) in this work computes the decision interval based on the transformation of standardized renting/returning demand. To reduce the risk of over-repositioning, the *demand transformation* (DT) would refer to later demands but it is impracticable to a dynamic repositioning strategy. Since several works exhibit hourly and daily time series periodicity in historical bike-sharing demand, we use past records instead and the following renting and returning DT formulas, where *C_s_* is the station capacity and *w* ∈ (0, 1) is the given weight.

(3) $$DT_{rent}\left(C_{s},T,c,w\right)=\frac{1}{2}\left(\overset{T}{\underset{i=0}{\sum }}w{\left(1-w\right)}^{i}\times DS_{rent}\left(C_{s},t_{i},c\right)\right)$$

(4) $$DT_{return}\left(C_{s},T,c,w\right)=\frac{1}{2}\left(\overset{T}{\underset{i=0}{\sum }}w{\left(1-w\right)}^{i}\times DS_{return}\left(C_{s},t_{i},c\right)\right)$$

The effect of later demands in the formula exponentially decay over time owing to the weight *w*, while the summation of weights to all DS demands is 1. Then, we compute the decision interval for a station *s* where the targeted value *Target* (*C_s_*, *T*, *c*, *w*) is the sum of two DT formulas as follows. The lower and upper values comprise the interval according to the targeted value and the present demand is crucial. On account of a full station by returning bikes, we should control the upper value well, while the low value largely concerns the renting demand. In case a station has a large renting/returning demand at a certain period of time, we should walk on eggshells to keep the station available. By intuition, the lower or upper values should approach the targeted value during the rush hour demand for a rebalance of available stalls. Therefore, the formulas of lower and upper values are as follows:

(5) $$Target\left(C_{s},T,c,w\right)=DT_{rent}\left(C_{s},T,c,w\right)+DT_{return}\left(C_{s},T,c,w\right)$$

(6) $$Upper\left(C_{s},T,c,w,\;b\right)=Target+\left(1-Target\right)\times Gap\left(DS_{return},\;b\right)$$

(7) $$Lower\left(C_{s},T,c,w,\;b\right)=Target-Target\times Gap\left(DS_{rent},\;b\right)$$

(8) $$Gap\left(x,\;b\right)=\frac{2\left(1-b\right)}{1+{\left(\frac{2}{b}-3\right)}^{2x}}$$

**Figure 8:**
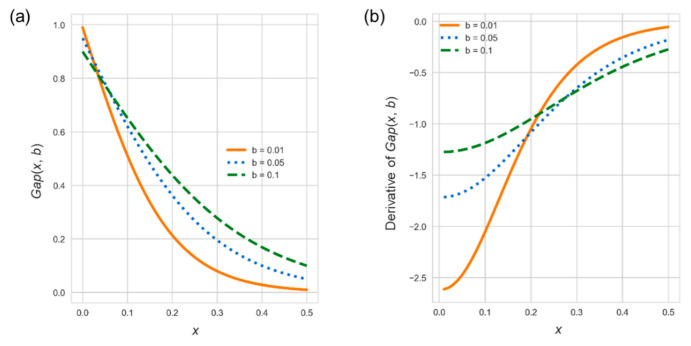
(**a**). *Gap*(*x*, *b*) curves under different *b* values; (**b**). Slopes of curves in (**a**).

The function *Gap*(*x*, *b*) is to regulate lower and upper values where *x* is in [0, 1] and the hyper-parameter *b* is the size of the gap to 0 and 1. There are two purposes for including the regulation function: First, a much lower demand would make lower and upper values close to 0 and 1, respectively, leading to start the repositioning operations in extreme states of almost empty or full stations. Therefore, the lower (resp. upper) value should have a gap to 0 (resp. 1). Second, a much higher demand would make lower and upper values both approach the targeted value, bringing on frequent repositioning operations. To reduce the repositioning cost, we should introduce a gap between lower/upper and targeted values. Figure 8 demonstrates *Gap*(*x*, *b*) curves for different *b* values. From Figure 8a, a larger *b* in *Gap*(*x*, *b*) leaves a greater gap to both 0 and 1. Figure 8b exhibits slopes of three curves in Figure 8a, while the three slopes are initially negative and close to 0 as *x* increases, indicating that the curve is becoming smoother. In other words, we expect that both lower and upper values quickly move far away from the targeted value at low demand times, and as the standardized renting (resp. returning) demand increases, the lower (resp. upper) value slowly approaches 0 (resp. station capacity), i.e., an empty (resp. full) station.

## 3. Results

### 3.1. Cluster Visualization

According to the insights from Section 2.2, we know that the demand patterns in days are different, and thus there are four categories, i.e., dull and peak seasons with each having two subgroups of working and non-working days, to be examined. For each station in a category, we individually compute averages of aggregated RRDs within 30 min and standardize them by *DS_rent_* and *DS_return_*. Then, a station is represented by 48 features where each is *DS_return_*(*t_i_*) − *DS_rent_*(*t_i_*) of the time *t_i_*. We employ the *k*-means clustering with the Euclidean distance for stations in each category, while two succinct graphical representations of silhouette and elbow illustration are utilized to measure how well each station has been classified. As a result, we determine appropriate values of cluster *k* for the four categories individually from prior knowledge of graphical representations and examine their difference further.

**Figure 9:**
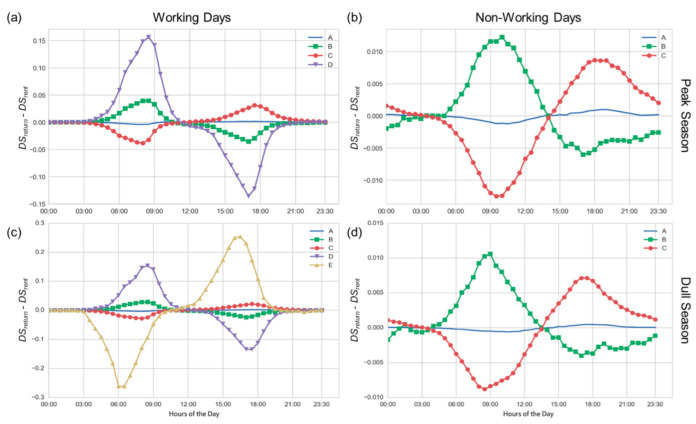
(**a**–**d**) show cluster centroids for the four categories whose name is next to figures.

Figure 9 demonstrates cluster centroids for the four categories. Stations in dull seasons and on working days are partitioned into four clusters, in which each station belongs to the cluster with the nearest cluster centroids shown by Figure 9a. The smooth curve of cluster A may be caused by two cases: One is generally in low demand for most stations in this cluster. The other is that RRDs are almost equal all the time and thus it approaches 0 after the subtraction. However, we hardly observe similar RRDs within 30 min from the dataset except for low-demand times. Therefore, we mark the A cluster as “Stable”, which appears in the four categories with different cluster sizes. Table 1 reveals that the number of stations in four A clusters is certainly the maximum among clusters in the same category and has the lowest averages of station capacities as well as the sum of RRDs for these clusters. In other words, low-demand stations make up a large proportion of the whole.

Clusters B and C have opposite demand types, where the former prefers returning bikes in the morning and renting bikes in the afternoon. Stations in cluster B are probably installed near activity centers or attractions, whereas cluster C stations are perhaps close to residential districts. The two cluster centroids have much alike patterns but with different scales, where two clusters on working days (Figure 9a,c) and on non-working days (Figure 9b,d) are similar. From Table 1, two clusters among four categories have similar characteristics within their category; even so, they are slightly different due to the effect of two seasons. For example, cluster B is higher than cluster C in both averages of station capacities and sum of RRDs, while the average sums of RRDs of two clusters on working days are significantly lower than on non-working days regardless of season type. However, with respect to average sums of RRDs, clusters B and C in peak seasons are apparently higher than in dull seasons, respectively.

As for cluster D in Figure 9a, its demand pattern is similar to cluster B but with a more dramatic variation, as the cluster D in Figure 9c. Stations in cluster D are in high demand and have the greatest averages of station capacities in the two categories (Table 1), likely deployed at business districts. Compared with clusters A, B and C, the number of stations in cluster D is quite small, that is, there are excessive demands in a few bike stations during rush hours. Moreover, cluster E contains only one station whose demand pattern is contrary to the centroid of cluster D illustrated in Figure 9c. This station is particular due to its small capacity and high-demand requirement.

**Figure 10:**
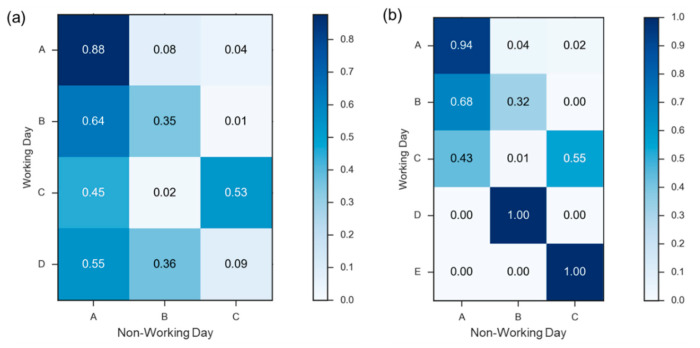
Comparison of cluster stations between working and non-working days in (**a**) peak and (**b**) dull seasons.

Figure 10 exhibits the shift of cluster members between working and non-working days in two seasons where the value in each square is the shift percentage of stations from the working to non-working day. For instance, the pair (A, A) in Figure 10a represents that 88% stations changed from cluster A on working days to the same cluster on non-working days, that is, the demands of these stations in peak season are quite similar regardless of two considered types of days. Additionally, 8% and 4% of stations in cluster A on working days regrouped into clusters B and C on non-working days, respectively. As illustrated by Figure 10a, over half of the stations in four clusters of working days are changed to cluster A of non-working days, indicating that the demand is becoming low in many stations. Stations in the same cluster on two types of day suggest a consistent demand type. On the other hand, Figure 10b demonstrates the comparison of cluster members of two types of day in the dull season. Similar to Figure 10a, many stations in clusters A, B and C on working days belong to cluster A on non-working days. High-demand stations in clusters D and E on working days become low-demand stations on non-working days, and thus they are partitioned into clusters B and C, respectively. On account of the inconspicuous demand for stations on non-working days, our repositioning simulations are only for working days in two seasons.

**Figure 11:**
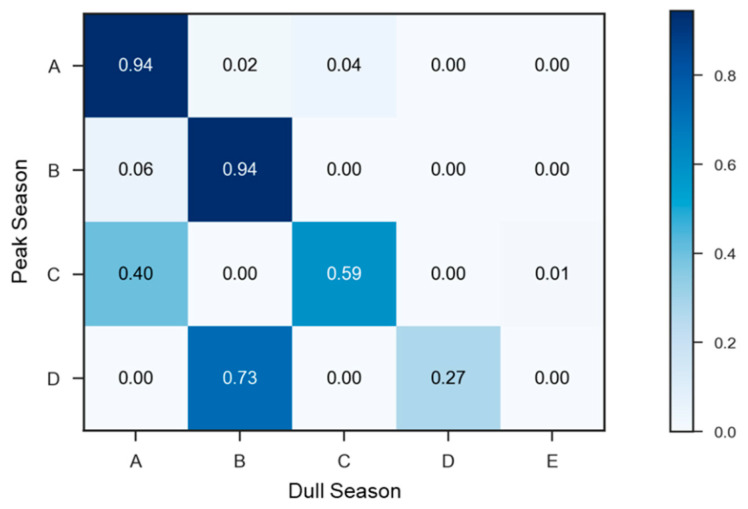
Comparison of station clusters in working days of peak and dull seasons.

Moreover, we examine the shift of cluster members on working days between peak and dull seasons in Figure 11. Most stations in clusters A and B in peak season are in the same clusters in the dull season. A total of 40% and 1% stations in cluster C in the peak season change to clusters A and E in the dull season, respectively. Only 27% of stations are in cluster D in both peak and dull seasons, which have a high demand irrespective of season type, and the others (73%) have a low demand in the dull season due to a shift of the cluster from D to B. On the whole, the daily demand for stations in the peak season is about five times larger than in the dull season, implying that stations in the dull season hardly have peak demands as in the peak season. As a result, many stations in clusters C and D in peak season change to clusters A and B with lower demands in the dull season, respectively.

### 3.2. Performance Measurement

We need several measurements to disinterestedly evaluate the performance of a repositioning strategy. Apart from the measurements in [17], we also develop several indicators in order to obtain a comprehensive evaluation of our repositioning approach. At first, we introduce two measurements proposed by Hulot et al. [17]. We assume that the number of stalls is in the repositioning interval, the worst cases when a station is snowed under with renting or returning workloads are computed individually, and thus a station has two values in the following where *d*(*s*, *t*) and *d*(*s*, *t*) are the number of lost bike rentals (departures) and returns (arrivals) of station s during period t, respectively. Two notations of *I_min_*(*s*, *t*) and *I_max_*(*s*, *t*) are the lower and upper bounds of a decision interval, respectively.

(9) $$lost_{dep}=mean_{s\in S,t\in T}max\left(0,\left(d\left(s,t\right)-I_{min}\left(s,\;t\right)\right)\right)$$

(10) $$lost_{arr}=mean_{s\in S,t\in T}max\left(0,\left(a\left(s,t\right)-\left(C_{s}-I_{max}\left(s,\;t\right)\right)\right)\right)$$

The average number of lost departures and arrivals are used for the score of lost trips from the worst-case point of view. For example, we can look at a station *s* with maximum capacity 10 (*C_s_* = 10) and a decision interval of [5,8] during period *t*. If there are seven bikes for departure and one for arrival, then *lost_dep_* = *max*(0, (7 – 5)) = 2 and *lost_arr_* = *max*(0, 1 – (10 – 8)) = 0. Both scores are good with a small value in the worst-case analysis, and thus a well-functioning repositioning strategy is supposed to minimize the two scores as much as possible. Furthermore, a narrower decision interval for a station keeps it in good condition but results in frequent repositioning operations and high cost. Therefore, we adopt two notations, *alert*^+^ and *alert*^−^, to denote the accumulative numbers beyond the upper and lower bounds for a station, respectively, and compute them according to the RRDs for each period *t* in the simulation introduced later. Once the station demand is beyond the decision interval, our strategy starts the repositioning operation. That is, *alert*^+^ and *alert*^−^ also represent the accumulative numbers of restoring available bikes and stalls, respectively, in each station to its targeted value by transporting vehicles. In addition to the number of transporting bikes, the number of bikes refilled and removed, denoted as *rebalance*^+^ and *rebalance*^−^, are considered.

### 3.3. Simulation Result

We conducted simulations for our DCRS strategy and compared it with the repositioning approach of Hulot et al. [17], denoted as SL, whose primary idea is the so-called *service level* as the expected satisfied demand over the expected total demand [28]. A station has rental and return service levels based on its maximum capacity, expected RRDs and the distribution of trips. SL has two hyper-parameters, *α* and *β*, where the former is to prefer either returns or rentals and the former indicates how exigent the operator is about the system, while we utilize the suggested values as [17] in the simulation.

The simulation of our DCRS is based on the historical datasets with resampling of the number of trips by 30 min. Algorithm 1 is the pseudocode for DCRS simulation at a station where Lines 1–7 set initial values. Line 1 returns decision intervals of a station in all periods, Line 2 sets the initial targeted value as the original number of stalls, and Line 3 assigns the sum of rentals and returns. Lines 4–7 perform initialization for seven measurements. There is a loop in Lines 8–20 to update measurements one by one. If the current number of bikes is smaller than the lower boundary, then the DCRS operation transports bikes to the station and updates the corresponding two measurements; on the contrary, Lines 17–20 consider the case when an excessive number of bikes comes about. Additionally, the four scores are highly dependent on the size of the decision interval: a narrower interval will get smaller *lost_dep_* and *lost_arr_* but greater *alert*^+^ and *alert*^−^. Therefore, repositioning strategies are supposed to have approximate interval sizes for the comparison.
**Algorithm 1.** Pseudocode for DCRS simulation and performance measurementsParameters:*C_s_*: The Maximum Capacity of a Station *s*Decision_Interval(*s*): The Decision Interval of a Station *s*Target (*t*_0_): The Targeted Value at Time *t*_0_1:Low, Target, Up = Decision_Interval(*s*)2:num_bikes = Target[t_0_]3:net_traffic = arrivals − departures4:*lost_dep_*, *lost_arr_* = 0, 05:*alert*^+^, *alert*^−^ = 0, 06:*rebalance*^+^, *rebalance*^−^ = 0, 07:interval_size = 08:**for** each time *t* **do**9:    num_bikes += net_traffic[*t*]10:    *lost_dep_* = max(0, num_bikes − Low[*t*])11:    *lost_arr_* = max(0, num_bikes + Up[*t*] − *C_s_*)12:    interval_size[*t*] = Up[*t*] − Low[*t*]13:    **if** num_bikes < Low[*t*] **then**14:       *alert*^+^ += 115:       *rebalance*^+^ += Target[*t*] − num_bikes16:       num_bikes = Target[t]17:    **else if** num_bikes > Up[*t*] **then**18:       *alert*^−^ += 119:       *rebalance*^-^ += num_bikes − Target[*t*]20:       num_bikes = Target[t]

As mentioned previously, stations on non-working days of two considered seasons have a low demand, and thus there are seldom repositioning requirements. For these stations, a static repositioning scheme is usually better than a dynamic approach. Therefore, we conduct repositioning simulations for stations on working days of two seasons and reveal the performance measurements in Table 2 and Table 3. Firstly, Table 2 summarizes several scores for two repositioning strategies to the four clusters of the peak season, where SL is the work of Hulot et al. [17]. As there is a similar interval size in two repositioning strategies, the comparison of six measurements is instructive. For the four clusters, our DCRS is remarkably smaller than SL in *lost_dep_* and *lost_arr_*, indicating the excellent performance of DCRS in the worst case. Two mean numbers of repositioning operations (*alert*^+^ and *alert*^−^) of two strategies in four clusters are approximate; however, DCRS works well in cluster D of high demand since it has lower mean numbers of transporting bikes (*rebalance*^+^ and *rebalance*^−^), indicating that DCRS can substantially reduce the CO_2_ emission and repositioning cost. Compared with SL, DCRS has an outstanding performance on working days of the peak season.

For the five clusters in the dull season, Table 3 exhibits the repositioning performance of DCRS and SL. The interval sizes of two repositioning strategies of these clusters, except cluster E, are close, while in two measurements of *lost_dep_* and *lost_arr_*, DCRS is also better than SL in the four clusters. With respect to clusters A and C, a slightly higher *rebalance*^+^ and *rebalance*^−^ of DCRS than SL implies a bit more of a repositioning cost; however, it greatly reduces the number of lost trips when the worst-case scenario occurs. Clusters D and E have a high demand and DCRS is considerably better than SL in the former from Table 3. The comparison in cluster E is questionable due to not only the different interval sizes but also one station in the cluster. The larger interval size of DCRS indeed obtains a smaller average number of repositioning operations, while smaller *lost_arr_* and *rebalance*^−^ of DCRS suggest the great efficiency compared to SL. However, DCRS works poorly in reducing the number of lost departures, which perhaps results from the contradiction between the small capacity and high demand of this station.

## 4. Conclusions

The smart city integrates ICT and various physical IoT devices to enhance the efficiency of city operations and deliver urban services, where the BSS is indispensable to reduce costs and resource consumption for developing smart urban services. In this work, we explore two open datasets, Citi Bike trip and transportation data, and investigate the demand patterns of rentals and returns. By the insight of historical demands from dataset, the trip times are sophisticatedly divided into four categories corresponding to four combinations of two seasons and two days. Then, we developed a non-linear scaling technique to standardize the demands of rentals and returns by placing emphasis on demands higher than the station capacity. The cluster analysis is exploited to group stations with similar demand patterns into clusters and visualize the cluster centroids for each category. By the station clustering analysis, there are different numbers of clusters among the four categories, and, all things considered, the working and non-working days have distinct demand patterns whereas the peak and dull seasons have similar demand patterns but with discrepant scales. In view of the repositioning operations for success of a BSS, we developed a repositioning scheme called DCRS on the basis of standardized demands and conducted the simulation on working days of two considered seasons due to the low demand on non-working days. Compared with the repositioning work of Hulot et al., our DCRS strategy has a better outcome through several performance measurements. In the peak season, DCRS can not only keep stations available by a smaller number of repositioning operations but also reduce lost trips from the worst-case analysis; in the dull season, DCRS can also effectively reduce lost trips to all clusters, except one containing one station only. On the whole, DCRS performs well when the station demand is high regardless of season types and substantially reduces the CO_2_ emission and repositioning cost.

## Figures and Tables

**Table 1:** Comparison of clusters for the four categories.

			Cluster Names				
			A (Stable)	B (MRtARn-LD ^1^)	C (MRnARt-LD)	D (MRtARn-HD)	E (MRnARt-HD ^2^)
Peak Season	Working day (Figure 9a)	Number of Stations	375	109	132	11	-
		Avg. of Station Capacities	29.09	35.15	32.64	43.54	-
		Avg. Sum of RRDs	125.30	272.58	213.12	436.83	-
	Non-working day (Figure 9b)	Number of Stations	465	74	88	-	-
		Avg. of Station Capacities	29.88	37.32	32.67	-	-
		Avg. Sum of RRDs	133.20	347.20	250.08	-	-
Dull Season	Working day (Figure 9c)	Number of Stations	413	118	92	3	1
		Avg. of Station Capacities	28.69	36.54	34.94	44.33	19
		Avg. Sum of RRDs	61.51	157.05	156.87	339.06	405.59
	Non-working day (Figure 9d)	Number of Stations	508	58	61	-	-
		Avg. of Station Capacities	30.07	38.24	33.40	-	-
		Avg. Sum of RRDs	73.32	205.46	174.15	-	-

^1^ MRtARn-LD is short for “Morning Return and Afternoon Rent at Low Demand”. ^2^ MRnARt-HD is short for “Morning Rent and Afternoon Return at High Demand”.

**Table 2:** Comparison for two repositioning strategies to the four clusters of the peak season.

Cluster Name	A (Stable)		B (MRtARn-LD)		C (MRnARt-LD)		D (MRtARn-HD)	
Strategy	DCRS	SL	DCRS	SL	DCRS	SL	DCRS	SL
*lost_dep_*	39.45	41.46	73.03	75.06	63.98	64.93	146.33	152.41
*lost_arr_*	20.47	39.61	49.71	80.26	31.87	56.06	109.45	159.36
mean *alert*^+^	0.82	0.85	2.60	2.73	2.25	2.24	7.54	8.27
mean *alert*^−^	0.81	0.82	2.36	2.45	2.26	2.24	6.38	6.86
mean *rebalance*^+^	10.72	10.62	38.69	38.11	32.18	32.27	156.35	160.98
mean *rebalance*^−^	11.07	10.98	34.18	33.53	36.21	36.38	149.17	153.68
mean interval_size	25.87	26.32	30.27	29.89	27.24	27.02	32.82	31.31

**Table 3:** Comparison of two repositioning strategies of the five clusters of the dull season.

Cluster Name	A (Stable)		B (MRtARn-LD)		C (MRnARt-LD)		D (MRtARn-HD)		E (MRnARt-HD)	
Strategy	DCRS	SL	DCRS	SL	DCRS	SL	DCRS	SL	DCRS	SL
*lost_dep_*	17.16	23.22	36.31	48.42	40.02	50.94	93.51	120.12	228.78	196.28
*lost_arr_*	7.03	22.32	21.96	53.36	17.65	45.02	75.59	127.62	169.61	181.81
mean *alert*^+^	0.37	0.35	1.47	1.39	1.34	1.22	6.13	6.20	11.57	14.97
mean *alert*^−^	0.40	0.38	1.28	1.17	1.43	1.33	5.51	5.11	10.10	12.69
mean *rebalance*^+^	4.61	4.51	23.11	23.13	21.42	21.35	135.95	141.16	184.34	193.69
mean *rebalance*^−^	5.26	5.17	19.26	19.26	24.19	24.18	120.73	125.88	176.68	185.98
mean interval_size	26.04	27.38	32.24	33.19	29.72	30.36	35.22	34.46	13.16	10.10

## Data Availability

All data has been included in the study.

## References

[1] Alavi A.H., Jiao P., Buttlar W.G., Lajnef N. (2018). Internet of Things-Enabled Smart Cities: State-of-the-Art and Future Trends. Measurement.

[2] Sánchez-Vergara J.I., Ginieis M., Papaoikonomou E. (2021). The Emergence of the Sharing City: A Systematic Literature Review to Understand the Notion of The Sharing City and Explore Future Research Paths. J. Clean. Prod..

[3] Eren E., Uz V.E. (2020). A Review on Bike-Sharing: The Factors Affecting Bike-Sharing Demand. Sustain. Cities Soc..

[4] Faghih-Imani A., Eluru N. (2016). Incorporating the Impact of Spatio-Temporal Interactions on Bicycle Sharing System Demand: A Case Study of New York Citibike System. J. Transp. Geogr..

[5] Morton C. (2020). The Demand for Cycle Sharing: Examining the Links between Weather Conditions, Air Quality Levels, and Cycling Demand for Regular and Casual Users. J. Transp. Geogr..

[6] Chiariotti F., Pielli C., Zanella C., Zorzi M. (2018). A Dynamic Approach to Rebalancing Bike-Sharing Systems. Sensors.

[7] Ma X., Ji Y., Yuan Y., Oort N.V., Jin Y., Hoogendoorn S. (2020). A Comparison in Travel Patterns and Determinants of User Demand between Docked and Dockless Bike-Sharing Systems Using Multi-Sourced Data. Transp. Res. Part A Policy Pract..

[8] Hu S., Xiong C., Liu Z., Zhang L. (2021). Examining Spatiotemporal Changing Patterns of Bike-Sharing Usage during COVID-19 Pandemic. J. Transp. Geogr..

[9] Xinwei M., Ruiming C., Yuchuan J. (2019). Spatiotemporal Clustering Analysis of Bicycle Sharing System with Data Mining Approach. Information.

[10] Díaz J.J.V., Pozo R.F., González A.B.R., Wilby M.R., Ávila C.S. (2020). Hierarchical Agglomerative Clustering of Bicycle Sharing Stations Based on Ultra-Light Edge Computing. Sensors.

[11] Fontes T., Arantes M., Figueiredo P.V., Novais P. Bike-Sharing Docking Stations Identification Using Clustering Methods in Lisbon City. Proceedings of the 18th International Symposium on Distributed Computing and Artificial Intelligence.

[12] Fontes T., Arantes M., Figueiredo P.V., Novais P. (2022). A Cluster-Based Approach Using Smartphone Data for Bike-Sharing Docking Stations Identification: Lisbon Case Study. Smart Cities.

[13] Huang J., Tan Q., Li H., Li A., Huang L. (2022). Monte Carlo Tree Search for Dynamic Bike Repositioning in Bike-Sharing Systems. Appl. Intell..

[14] Wang X., Sun H., Zhang S., Lv Y., Li T. (2022). Bike Sharing Rebalancing Problem with Variable Demand. Phys. A.

[15] Bordagaray M., Dell’Olio L., Fonzone A., Ibeas A. (2016). Capturing the Conditions That Introduce Systematic Variation in Bike-Sharing Travel Behavior Using Data Mining Techniques. Transp. Res. Part C Emerg. Technol..

[16] Liu X., Pelechrinis K. (2021). Excess Demand Prediction for Bike Sharing Systems. PLoS ONE.

[17] Hulot P., Aloise D., Jena S. Towards Station-Level Demand Prediction for Effective Rebalancing in Bike-Sharing Systems. Proceedings of the 24th ACM SIGKDD International Conference on Knowledge Discovery & Data Mining.

[18] Collini E., Nesi P., Pantaleo G. (2021). Deep Learning for Short-Term Prediction of Available Bikes on Bike-Sharing Stations. IEEE Access.

[19] Boufidis N., Nikiforiadis A., Chrysostomou K., Aifadopoulou G. (2020). Development of A Station-Level Demand Prediction and Visualization Tool to Support Bike-Sharing Systems’ Operators. Transport. Res. Proc..

[20] Qiao S., Han N., Huang J., Yue K., Mao R., Shu H., He Q., Wu X. (2021). A Dynamic Convolutional Neural Network Based Shared-Bike Demand Forecasting Model. ACM Trans. Intell. Syst. Technol..

[21] Tuli F.M., Mitra S., Crews M.B. (2021). Factors Influencing the Usage of Shared E-Scooters in Chicago. Transp. Res. Part A Policy Pract..

[22] Kou Z., Cai H. (2019). Understanding bike sharing travel patterns: An Analysis of Trip Data from Eight Cities. Phys. A.

[23] Yan Q., Gao K., Sun L., Shao M. (2020). Spatio-Temporal Usage Patterns of Dockless Bike-Sharing Service Linking to A Metro Station: A Case Study in Shanghai, China. Sustainability.

[24] Alaoui E.A.A., Tekouabou S.C.K. (2021). Intelligent Management of Bike Sharing in Smart Cities Using Machine Learning and Internet of Things. Sustain. Cities Soc..

[25] Cruz F., Subramanian A., Bruck B.P., Iori M. (2017). A Heuristic Algorithm for A Single Vehicle Static Bike Sharing Rebalancing Problem. Comput. Oper. Res..

[26] Ren Y., Meng L., Zhao F., Zhang C., Guo H., Tian Y., Tong W., Sutherland J.W. (2020). An Improved General Variable Neighborhood Search for a Static Bike-Sharing Rebalancing Problem Considering the Depot Inventory. Expert Syst. Appl..

[27] Contardo C., Morency C., Rousseau L.-M. (2012). Balancing a Dynamic Public Bike-Sharing System.

[28] Schuijbroek J., Hampshire R.C., Hoeve W.-J.V. (2017). Inventory Rebalancing and Vehicle Routing in Bike Sharing Systems. Eur. J. Oper. Res..

[29] Legros B. (2019). Dynamic Repositioning Strategy in A Bike-Sharing System; How to Prioritize and How to Rebalance a Bike Station. Eur. J. Oper. Res..

[30] Vallez C.M., Castro M., Contreras D. (2021). Challenges and Opportunities in Dock-Based Bike-Sharing Rebalancing: A Systematic Review. Sustainability.

[31] Hu Z., Huang K., Zhang E., Ge Q., Yang X. (2021). Rebalancing Strategy for Bike-Sharing Systems Based on the Model of Level of Detail. J. Adv. Transp..

[32] Cipriano M., Colomba L., Garza P. (2021). A Data-Driven Based Dynamic Rebalancing Methodology for Bike Sharing Systems. Appl. Sci..

[33] Mehdizadeh Dastjerdi A., Morency C. (2022). Bike-Sharing Demand Prediction at Community Level under COVID-19 Using Deep Learning. Sensors.

[34] Ma X., Yin Y., Jin Y., He M., Zhu M. (2022). Short-Term Prediction of Bike-Sharing Demand Using Multi-Source Data: A Spatial-Temporal Graph Attentional LSTM Approach. Appl. Sci..

[35] Citi Bike System Data. https://ride.citibikenyc.com/system-data.

[36] Tyndall J. (2018). Bus Quality Improvements and Local Commuter Mode Share. Transp. Res. A Policy Pract..

[37] The Open Bus. https://www.theopenbus.com.

[38] Federal Holidays in New York in 2018. https://www.officeholidays.com/countries/usa/new-york/2018.

